# Prolapse of the Ovaries—Pelvic Hæmatocele

**Published:** 1880-12-20

**Authors:** Wm. Goodell


### PROLAPSE OF THE OVARIES—PELVIC HÆMATOCELE.

A CLINICAL LECTURE BY WM. GOODELL, M.D.

*Prolapse of the Ovaries.*—The ovary is a follicular gland, and its normal size and shape is about that of an almond. These ovaries are congested at every menstrual period. It may very properly be said that a woman is a woman because she has ovaries, and not because she has a womb. Many diseases of the womb can be traced to the ovaries. The symptoms of a prolapse of one or both of the ovaries are somewhat as follows: the woman has pain in the region of the womb and tenderness in the groins. The physician will notice a small round tumor in her vagina, which gives rise to a most sickening sensation when pressed. These tumors may vary in size, all the way from the normal size of the ovary to that of a large apple. When a prolapsed ovary is of very great size, there has usually been some cystic degeneration.

This accident (prolapse) is the opprobrium of gynecologists. Thus far no positive and permanent cure has been discovered. Floating about, as the ovaries do, in the atmosphere of the abdomen, the only wonder is that they do not prolapse more frequently. The abdomen, however, is so tightly packed with the viscera, that dislocations of the organs contained in it are quite rare.

The treatment of prolapsed ovaries is, as I have just remarked, often exceedingly difficult. The worst features of the condition are generally the mental symptoms. We may put our patient in bed and subject her to a routine treatment by massage and galvanism. Ovarian troubles very often depend upon some defect of nutrition, the best remedy for which is that set forth in Dr. S. Weir Mitchell's book on "Fat and Blood, and How to Make Them." I have obtained the most remarkable cures by the continued employment of the different items of this treatment. In this case there is no cystic degeneration; the ovaries have simply become congested and fallen behind the womb and into Douglas' pouch by their own weight. There is another cause of inflammation of the ovaries in which treatment is hopeless. Such is the case where ovaritis has been brought on by gonorrhœal poison. This infection runs through the mucous membrane of the vagina and womb, and finding its way out through the Fallopian tubes, brings on peritonitis, ovaritis, or pelvic cellulitis. A woman suffering from ovaritis may be permanently sterile. This is only the case, of course, where both ovaries are diseased. If one ovary escapes, the woman may become pregnant, and so obtain a rest for a space of nine months, during which time the other ovary may be able to regain its health.

What is to be done in this case? As far as medicines are concerned,

I shall order the patient ten grains of the muriate of ammonia, with one-twelfth of a grain of the bichloride of mercury, thrice daily. The muriate of ammonia may also be given with the mistura glycyrrhizæ composita. The antimony and paregoric in it are also very excellent remedies. I sometimes give small doses of tincture of aconite, two to five drops at a time. In this case the patient has been very much relieved by the use of pessaries (pessaries generally give great pain). Indeed, I think the patient has escaped very easily. The bend of the retroflexed womb has acted as a prop and prevented this organ from squeezing the prolapsed ovaries.

What is to be said as to the performance of ovariotomy for the relief of this prolapse? The operation would be easy, owing to the position of the ovaries, but the small amount of pain and the general good condition of the patient, thus far, render ovariotomy entirely unnecessary. The hard-rubber pessary which I have used has acted as a shelf to hold them up. An examination in this case shows me that the cervix is slightly lacerated, and that there is a slight erosion of the mucous membrane; the fundus of the womb, too, is very tender. I will treat these conditions by an application of carbolic acid.

*Hæmatoma in Douglas' Pouch.*—This woman is forty-two years old and married. She has had no children, and has suffered from a great deal of dysmenorrhœa at each monthly period. Now what do these things suggest? A flexion of the neck of the womb, of course. She tells me that she has been suffering in this way ever since she was seventeen years old. Early in her eighteenth year she consulted a physician for her dysmenorrhœa, and he did something to enlarge her cervical canal. The operation greatly relieved her. She says, by the way, that she menstruates every three weeks. Just about three weeks ago she had, one day, a violent pain across her stomach. Soon after this was a strong desire to defecate, which could not be satisfied. Since that time she has had constant pain, and has always felt a tumor internally. She came to me as a case of tumor of the womb. I am inclined, at first sight and without questioning the woman, to doubt that statement, and rather consider the case one of pelvic cellulitis or pelvic peritonitis. The text-books say that these are two distinct diseases, but I am led to think them, if not one and the same disease, at least very hard to separate. The cellulitis is very likely to, in fact, always does, involve the neighboring folds of the peritoneum. From what the woman tells me, I am inclined to put my first suspicions entirely out of the question and say that I believe the patient has had a hemorrhage into Douglas' pouch; that immediately after the hemorrhage the pouch bulged and so gave rise to the desire to defecate. She was sick a day or two after this pain, and the flow was very abundant. I can feel the tumor on the right side, and can move it slightly. Let me make a rectal and vaginal examination. First, let me pass my finger into her vagina. I feel two tumors, one behind and one before. The tumor in front is not painful. The diagnosis is puzzling. I think the tumor in front is the womb. I can very easily determine this point by passing a sound. Yes, my sound passes in and shows the womb to be of normal size. But how about the other tumor? What is that? I can move it much more than I could were it cellulitis. Undoubtedly there has been an effusion of blood into Douglas' pouch, which effusion has produced a slight pelvic peritonitis. When blood is effused into and confined by a shut cavity, the peritonitis or septicæmia is never so universal as it otherwise would be. By passing my finger into the rectum I am able still more clearly to fix the seat of the tumor, and at the same time note the fact that the rectum is greatly flattened. It requires some force to pass my finger through the flattened portion; now I have done so, and I find the tumor filling the position of the pouch, hard and immovable. We know that Douglas' pouch is lower on the left than on the right. In exact accordance with this fact I find that the tumor is lower on the left than the right side. Pelvic hæmatocele is sometimes exceedingly serious. What is the cause of it? At the same time of the monthlies the Graafian follicle may burst before the fimbriated extremity of the Fallopian tube is in relation to receive it, and so blood escapes into the abdomen and gravitates into the pouch. Or there may have been a retention of fluid at the monthlies which was forced from the womb into the Fallopian tube, and so out to Douglas' pouch. Sometimes, you know, extra-uterine fœtation occurs; in fact, there may be hemorrhage into Douglas' pouch from many causes. I believe that hæmatoma in Douglas' pouch occurs frequently without our knowledge.

How are we to treat this pelvic hæmatocele? It may happen, as I have just said, at a monthly period, and is attended with pain and collapse. Of course, we must make a viginal or rectal examination as soon as we see the case. We find, perhaps, an obscure fluctuation in Douglas' pouch; the blood is lying loose there, and has not yet, it may be, entirely congealed. The patient must be kept absolutely quiet, and astringent drinks, such as sulphuric acid, lemonade, etc., administered. Opium enough should be given to lull the pain and keep the patient thoroughly quiet. For a number of hours following the attack very slight nourishment should be given. Stimulants should be refrained from, and the patient kept as low as possible until all immediate danger from peritonitis has passed away. If the woman is married, her vagina should be packed with ice; if a virgin, the ice had better be placed outside.—*Clinical Record.*

---

**Acute Tonsillitis.**—We extract from article in Western Lancet the following: "I cannot too earnestly impress upon you the value of free scarification in cases of this kind. It is a simple procedure which at once relieves the dangerous congestion and inflammation, and affords the patient decided comfort. All that is necesssary to be done is to take a sharp-pointed instrument, like a tenotome or bistoury, and puncture the parts or make a few superficial incisions. Having depleted the parts thoroughly, we then make free use of ice, both internally and externally.

**Night-Sweats.**—A writer in the Thearapeutic Gazette says: "The latest remedy for the night-sweating of phthisis is muscarine, obtained from the agaricus muscarius, fly agaric or fly fungus. It is said to answer admirably. There are few better remedies than picrotoxine taken in the form of pills, gr. 1.60 every night at bed-time.

